# Arctic marine forest distribution models showcase potentially severe habitat losses for cryophilic species under climate change

**DOI:** 10.1111/gcb.16142

**Published:** 2022-03-08

**Authors:** Trevor T. Bringloe, David P. Wilkinson, Jesica Goldsmit, Amanda M. Savoie, Karen Filbee‐Dexter, Kathleen A. Macgregor, Kimberly L. Howland, Christopher W. McKindsey, Heroen Verbruggen

**Affiliations:** ^1^ School of BioSciences University of Melbourne Melbourne Victoria Australia; ^2^ Fisheries and Oceans Canada Arctic and Aquatic Research Division Winnipeg Manitoba Canada; ^3^ Fisheries and Oceans Canada Maurice Lamontagne Institute Mont‐Joli Québec Canada; ^4^ 6323 Centre for Arctic Knowledge and Exploration Canadian Museum of Nature Ottawa Ontario Canada; ^5^ Département de Biologie ArcticNet Québec Océan Université Laval Québec Québec Canada; ^6^ School of Biological Sciences UWA Oceans Institute University of Western Australia Crawley Western Australia Australia; ^7^ Institute of Marine Research Floedivigen Research Station His Norway

**Keywords:** ecological niche modelling, kelp, MaxEnt models, seaweed

## Abstract

The Arctic is among the fastest‐warming areas of the globe. Understanding the impact of climate change on foundational Arctic marine species is needed to provide insight on ecological resilience at high latitudes. Marine forests, the underwater seascapes formed by seaweeds, are predicted to expand their ranges further north in the Arctic in a warmer climate. Here, we investigated whether northern habitat gains will compensate for losses at the southern range edge by modelling marine forest distributions according to three distribution categories: cryophilic (species restricted to the Arctic environment), cryotolerant (species with broad environmental preferences inclusive but not limited to the Arctic environment), and cryophobic (species restricted to temperate conditions) marine forests. Using stacked MaxEnt models, we predicted the current extent of suitable habitat for contemporary and future marine forests under Representative Concentration Pathway Scenarios of increasing emissions (2.6, 4.5, 6.0, and 8.5). Our analyses indicate that cryophilic marine forests are already ubiquitous in the north, and thus cannot expand their range under climate change, resulting in an overall loss of habitat due to severe southern range contractions. The extent of marine forests within the Arctic basin, however, is predicted to remain largely stable under climate change with notable exceptions in some areas, particularly in the Canadian Archipelago. Succession may occur where cryophilic and cryotolerant species are extirpated at their southern range edge, resulting in ecosystem shifts towards temperate regimes at mid to high latitudes, though many aspects of these shifts, such as total biomass and depth range, remain to be field validated. Our results provide the first global synthesis of predicted changes to pan‐Arctic coastal marine forest ecosystems under climate change and suggest ecosystem transitions are unavoidable now for some areas.

## Graphical abstract

Climate change responses of Arctic marine forest species with respect to shifting habitat ranges may differ according to cold water preferences. Cryophilic species such as the red alga *Dilsea socialis* may contract their range northwards in the near future, with the amount of habitat lost depending on the severity of climate change. Photo credit for *Dilsea socialis*: Dr. Gary Saunders.

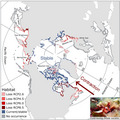

## 1 INTRODUCTION

Although climate change is a global phenomenon, some areas are more susceptible to warming trends. The Arctic, in particular, is warming at least twice as fast as the global average (Miller et al., 2010; IPCC, 2021), with October 2019–September 2020 representing the second warmest 12‐month period of observed surface air temperatures over Arctic land during the last century (Ballinger et al., 2020). Accelerated warming in the Arctic has even resulted in decoupling from Pleistocene climatic cycles, with the next glaciation no longer predicted to occur within the next 50 thousand years (Berger et al., 2016). Changes in the Arctic observed today will, therefore, be long‐term and likely irreversible (IPCC, 2021). These changes are occurring now, with declining perennial ice cover, ocean acidification, altered circulation patterns, and changes to sea‐surface temperatures and salinity (Renaud et al., 2015; Stroeve et al., 2012; Thornalley et al., 2018). Understanding the impact of these changes on marine ecosystems, particularly foundation species (Dayton, 1972), will provide important context for the state of the Arctic under a warmer climate.

Marine forests are dynamic underwater seascapes formed by seaweeds, typically consisting of a canopy composed of Laminariales or Fucales (Phaeophyceae), and an understory of smaller brown, green and red algae, which together provide crucial habitat for coastal ecosystems worldwide (Bruno & Bertness, 2001; Shiel and Foster, 2006; Teagle et al., 2017). Marine forests additionally create food, foraging and nursery grounds for numerous fish and invertebrate species (Bégin et al., 2004; Teagle et al., 2017), and their role in carbon sequestration remains an area of active investigation (Krause‐Jensen & Duarte, 2016; National Academies of Sciences, Engineering, and Medicine, 2021; Ortega et al., 2019). Marine forests also provide significant economic opportunities. The services provided by kelp and associated flora/fauna are estimated to value $500,000–1,000,000 USD per kilometre of coastline (Filbee‐Dexter & Wernberg, 2019). Moreover, different species of seaweed provide variations in the three‐dimensional habitat structure amenable to different fauna (Ware et al., 2019). Thus, the combination of species making up marine forests has a large effect on associated communities, with different assemblages of marine forest species providing different biological characters to the overall ecosystem(s). Of concern, highly variable localized responses have amounted to a small global average decline in kelps (Krumhansl et al., 2016), and marine heatwaves threaten to extirpate cold‐adapted flora in favour of warm‐adapted turf‐forming species, with accompanying changes to ecosystem function (Filbee‐Dexter & Wernberg, 2019; Filbee‐Dexter et al., 2020; Pessarrodona et al., 2021; Vergés et al., 2016).

Conversely, the Arctic has been hypothesized to feature marine forest species expanding north under climate change (Campana et al., 2009; Krause‐Jensen et al., 2020; Krause‐Jensen & Duarte, 2014; Müller et al., 2009). The Arctic currently hosts extensive marine forests (Filbee‐Dexter et al., 2019; Lüning, 1990; Wilce, 2016), with a portion of species recently derived from adjacent cold‐temperate oceans following the last glaciation (~21 ka; Bringloe et al., 2020). An increasingly large proportion of endemic species and populations, however, are being revealed by DNA data, suggesting these marine forests have persisted through cycles of glaciation and are potentially adapted to high latitude conditions (Bringloe et al., 2020). This unique assemblage of macroalgae at high latitudes imbues Arctic marine forests with a “northern” character distinct from temperate assemblages (Wilce, 2016). The northern extent of Arctic marine forests is believed to be limited by light, ice scour, and the extent of multi‐year sea‐ice, with suitable substrate requirements further limiting marine forests in parts of the Western Canadian, Northern Alaskan, Siberian, and Laptev Sea Arctic coastlines (Filbee‐Dexter et al., 2019; Lee, 1973; Wilce & Dunton, 2014). The contracting northern ice‐pack is, therefore, expected to open new marine forest habitat in the coming decades through increasing light availability and reduced scour (Campana et al., 2009; Krause‐Jensen & Duarte, 2014), though turbidity effects from increased wave exposure and glacial meltwaters (Bonsell & Dunton, 2018; Traiger & Konar, 2017) and freshening from ice melt/increased runoff (Lind & Konar, 2017) could negate these effects for some species. Furthermore, cold‐water marine forests are expected to decline at their southern range edge (Assis et al., 2018a; Jueterbock et al., 2013, 2016). As such, it is reasonable to expect succession in marine forest composition in the Arctic from being dominated by endemic and cold‐tolerant species to temperate taxa (e.g. Hop et al., 2012), with accompanying shifts to overall ecosystem function.

**FIGURE 1:**
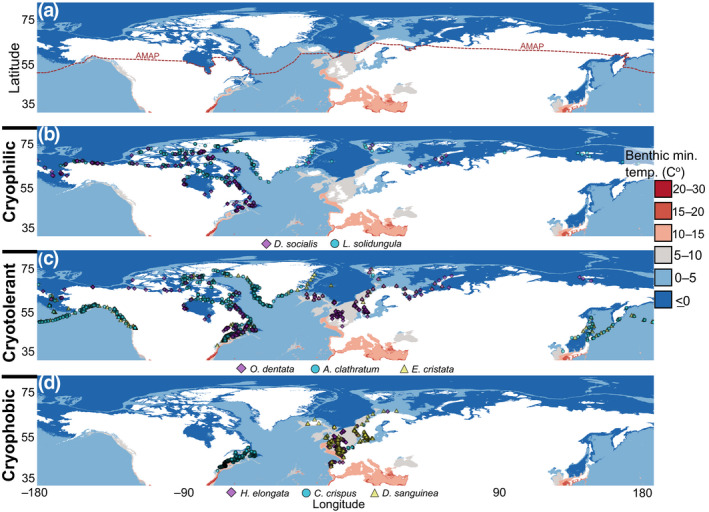
(a) Benthic minimum temperature regimes used to define the Arctic environment (<0°C), along with occurrence records used to train models of marine forest distributions under climate change according to affinity to the Arctic environment, including (b) cryophilic, (c) cryotolerant, and (d) cryophobic species. Records are overlaying Bio‐ORACLE layer benthic long‐term minimum annual temperature (C^°^; min. depth). Records are derived from Lüning (1990), digitized herbarium records (https://macroalgae.org/portal/; this study), among other databases (see methods). Records were further curated based on DNA barcode insights (Bringloe & Saunders, 2018; Bringloe et al., 2020). The red dashed line defines the Arctic basin, as per AMAP (1998)

A taxonomically broad, global analysis, comparing contemporary and projected distributions of marine forests under climate change, is needed to establish baseline distributions and quantify expected range shifts in species with different temperature preferences. Our objective was to estimate potential gains and losses in global northern hemisphere marine forests along the Arctic and temperate coastlines under different climate change scenarios. In recognizing the potential for Arctic endemic species to exhibit markedly different responses to shifting climate regimes compared to cold‐temperate taxa, we also sought to investigate anticipated gains and losses of habitat according to three distribution categories with varying affinities to the Arctic environment: cryophilic marine forest species (generally restricted to the Arctic environment; Figure 1b), widely distributed cryotolerant species (which occur in the Arctic to cold‐temperate environments; Figure 1c), and cryophobic species (generally restricted to cold‐temperate conditions with no current range in the Arctic environment; Figure 1d). We used stacked species distribution models to answer three research questions: (1) Will suitable marine forest habitat expand in the Arctic environment under climate change?; (2) Will potential habitat expansions at high latitudes compensate for losses in suitable habitat at the southern range edge?; (3) Will the above‐described categories feature similar or different responses in terms of gains and losses of habitat related to climate change? Addressing these questions will provide overarching insights on the responses of marine coastal systems to climate change, particularly with regards to the potential for high latitude shifts in marine forest services.

## 2 MATERIALS AND METHODS

Many definitions of the Arctic exist, along with the potential to generate considerable confusion. Thus, we first seek to clarify our definitions of the Arctic for the purpose of this study. In terms of geopolitical borders, we acknowledge the definition of AMAP (1998), which generally follows a 10°C July mean air isotherm that includes the Canadian coasts (including the Canadian Archipelago and Baffin‐Davis Straight) throughout the Hudson Complex and Labrador, as well as coastal areas in Greenland, Iceland, northern Norway, the entire Siberian coastline, the Bering Sea, and northern Alaska (Beaufort Sea; Figure 1a). For the purpose of analysing seaweed distributions, we define the Arctic environment as coastal areas with a long‐term annual benthic minimum seawater temperature <0°C (as averaged from 2000 to 2014; Assis et al., 2018b; Figure 1a), consistent with Adey and Steneck (2001). Benthic temperatures considered here correspond to the shallowest depth value from grids presented by the General Bathymetric Chart of the Oceans (2015), from which the minimum depth values of Bio‐ORACLE cells are derived (Assis et al., 2018b). Note, our definition of the Arctic environment differs from the distribution of AMAP; we accept that the Arctic environment from the perspective of marine forests includes some areas of the Canadian maritime provinces (e.g. see Edelstein et al., 1967), northern portions of the Sea of Okhotsk and Bering Sea, and parts of Svalbard whilst excluding mainland Norway, Iceland and the Aleutian Islands altogether (Figure 1a,b). We also recognize that this definition overlooks the influence of daylength on seaweed phenology, which varies considerably from mid to high latitudes, a point we address in the discussion.

### 2.1 Occurrence datasets

Eight species were selected for analysis on the basis of (1) being sublittoral and/or (to some extent) low littoral, (2) having a reasonable expectation of accurate species identification validated by DNA barcode surveys (e.g. Bringloe et al., 2020), and (3) having sufficient occurrence records for analysis. Occurrence records were sourced for macroalgal species falling under the categories reflecting affinity to the Arctic environment as defined above: (i) cryophilic: species restricted to waters with a long‐term minimum annual benthic temperature less than 0°C, including the kelp *Laminaria solidungula* J. Agardh, and the red alga *Dilsea socialis* (Postels & Ruprecht) Perestenko; (ii) cryotolerant: species occurring in, but not restricted to, areas with a long‐term minimum annual benthic temperature less than 0°C, including the kelp *Agarum clathratum* Dumortier, and the red algae *Euthora cristata* (C. Agardh) J. Agardh, and *Odonthalia dentata* (Linnaeus) Lyngbye; (iii) cryophobic: cold‐temperate species with a northward range limit corresponding to areas with a long‐term minimum annual benthic temperature exceeding 0°C, including the brown alga *Himanthalia elongata* (Linnaeus) S.F. Gray, and the red algae *Chondrus crispu*s Stackhouse and *Delesseria sanguinea* (Hudson) J.V. Lamouroux. For the occurrence dataset, records were first sourced by georeferencing distributions depicted in Lüning (1990) using ArcGIS. Note, the accuracy of these georeferenced records, whilst suitable for this study, may not be acceptable for modelling at higher spatial resolutions. For occurrences depicted as continuous along shorelines, occurrences were haphazardly georeferenced at approximate 200‐km intervals. These distributions summarize records from extensive historical surveys dating back to at least the late 19th century. Secondly, georeferenced records postdating 1900 were sourced from the Macroalgal Herbarium Portal (https://macroalgae.org/portal/index.php; downloaded 31 March 2020). Note the following synonyms were included in the searches: synonyms of *D*. *socialis*, including *Dilsea integra* (Kjellman) Rosenvinge, *Neodilsea integra* (Kjellman) A.D. Zinova and *Sarcophyllis arctica* Kjellman; synonyms of *E*. *cristata*, including *Callophyllis cristata* (C. Agardh) Kützing. Records for *D*. *socialis* and *O*. *dentata* from British Columbia (Canada), the Gulf of Alaska, and the Aleutian Islands were scrutinized and regarded as misidentifications or database annotation errors, and thus not included in our dataset. For *C*. *crispus*, a polygon was drawn around Atlantic records to exclude Pacific records (i.e., congeners). Occurrence records were also mined from the Barcode of Life Data Systems (http://v4.boldsystems.org//).

Additional occurrence data of *A*. *clathratum* and *L*. *solidungula*, were compiled from museum data records of kelp specimens from the National Herbarium of Canada at the Canadian Museum of Nature (CANA) together with data collected directly from research campaigns across the Eastern Canadian Arctic by taking pictures and videos of quadrats whilst diving (Filbee‐Dexter et al., In press). Biodiversity databases such as the Global Biodiversity Information Facility (GBIF ‐ www.gbif.org, accessed March 9th, 2020) and the Ocean Biodiversity Information System (OBIS; https://obis.org/) were further used for the two species of kelp (accessed 18 March 2020) and *H*. *elongata* (29 October 2021) and complemented with information from the literature (Filbee‐Dexter et al., 2019; Hop et al., 2016; Ronowicz et al., 2020; Schoenrock et al., 2018) and communications with experts (e.g. G. Saunders pers. comm.).

Records falling within the same environmental grid were counted as a single occurrence for model training, thus removing duplicates between datasets. Note that 88% of records post‐dated 1965, and 94% post‐dated 1950. For training the models, the number of geographically unique (i.e., non‐duplicate) records analysed was 91 for *D*. *socialis*, 153 for *L*. *solidungula*, 419 for *A*. *clathratum*, 300 for *E*. *cristata*, 136 for *O*. *dentata*, 403 for *C*. *crispus*, 69 for *D*. *sanguinea* and 173 for *H*. *elongata*. The dataset of all compiled occurrence records prior to deduplication is available via Figshare (https://doi.org/10.6084/m9.figshare.14751753.v6).

### 2.2 Modelling and area calculations

Present‐day surface and benthic environmental layers at minimum water depth were sourced from Bio‐ORACLEv2.1 (https://www.bio‐oracle.org/; Assis et al., 2018b), representing monthly averages for the years 2000–2014 (long‐term values). The spatial resolution of the environmental layers represented 5 arcmin or about 10 km^2^ at the equator. The layers included: bottom temperature (mean, maximum and minimum); bottom salinity (mean); surface ice thickness (mean, maximum and minimum). These layers were selected based on ecological/biological justifications. First, minimum depth benthic layers were selected (as opposed to surface, mean depth and max depth layers) given seaweeds are constrained by substrate and light requirements; given the coarse resolution of the environmental grids, we assumed the minimum depth benthic layers was most representative of biologically relevant conditions. Because the environmental grids can feature numerous depth values, the minimum depth refers to the shallowest measurement point in the grids, thus it is a dynamic rather than fixed value. Regarding environmental variables, ice thickness was included due to its biological relevance for cryophilic species (i.e., Arctic endemic species tune phenology to seasonal extremes in temperature and ice‐cover; e.g. Chapman & Lindley, 1981; Dunton & Schell, 1986). Temperature layers were included as seaweed distributions are commonly limited by lethal upper‐temperature limits, and decreased physiological performance/fitness at lower temperature preferences; in similar MaxEnt models of seaweed distributions, temperature is consistently among the most important variables driving distributions (e.g. Jueterbock et al., 2016; Martínez et al., 2018). Finally, mean salinity was selected, as this variable also occasionally explains a high percentage of seaweed distributions. Goldsmit et al. (2021), for instance, showed that ice thickness, temperature and salinity were the most important variables explaining kelp distributions in the Eastern Canadian Arctic. We reduced this layer selection further to remove layers with multicollinearity issues (selecting between the mean, long‐term maximum and long‐term minimum measures) using variance inflation factor (VIF) tests. We retained only the layers that had a VIF of <10 using the *usdm* R package (Naimi et al., 2014). Our final set of environmental layers was: minimum and maximum bottom temperature, mean bottom salinity and minimum and maximum ice thickness. Environmental layers for 2050 and 2100 under representative concentration pathways (RCP) 2.6, 4.5, 6.0 and 8.5 were also sourced from Bio‐ORACLEv2.1.

A mask of the study area was created (30°N and northwards) to only include cells of appropriate conditions that restrict the Arctic and temperate marine forest occurrences. First, cells were filtered to only include those whose centre point was within 10 km of the shoreline (the approximate resolution of the environmental grid at the equator) or had a depth between 0 and 100 m. Seaweeds are not expected at depths beyond 100 m (Krause‐Jensen et al. (2019) report kelp from 61 m off Greenland), though some crustose species of red alga are abundant in the Arctic (Peña et al., 2021) and may grow much deeper than this (Littler et al., 1985). The masking ensured cells that were too deep were excluded whilst retaining shoreline‐adjacent cells, where the mean depth at 5 arcmin resolution may exceed >100 m but includes some area of suitable depth (i.e., steep drop‐offs close to shore), and cells further offshore with a suitable depth (i.e., shallow coastal shelves). Second, as marine forests typically require a solid substrate, cells were further filtered to only include coastal areas identified as erosional (i.e., rocky), whilst excluding depositional coastal areas (i.e., muddy or sandy substrate), as per substrate results presented by Nyberg and Howell (2016).

All environmental layers were cropped to latitudes above 30°N, reprojected to the North Pole Lambert Azimuthal Equal Area projection, and then masked to our study area as described above. Each species was modelled independently via a MaxEnt model fit using the *dismo* R package (Hijmans et al., 2021). MaxEnt is a presence‐background modelling approach that minimizes the relative entropy between two probability densities in environmental covariate space: one estimated from a species’ presence records (where it is known to exist), and the other estimated from the background sample of the landscape (where it could possibly exist) (Elith et al., 2011). This provides insight on which covariates are important to a species and establishes the relative suitability of sites. MaxEnt has been shown to be amongst the top‐performing algorithms for presence‐background modelling (Valavi et al., 2022). Full details of our species distribution model methodology are reported following the overview, data, model, assessment, and prediction (ODMAP) protocol (Table S1; Zurell et al., 2020). Models were fitted using fivefold cross‐validation to tune the regularisation parameter of the model, as well as tuning the types of features used by the model to reduce overfitting. A model was then fitted to the full dataset of occurrence records for each species using these tuned parameters to make inferences and projections. For model evaluation purposes, we fit a separate fivefold spatial‐block cross‐validation run of the model (Valavi et al., 2019). We attempted to account for sampling bias in the presence‐only models using a target group background approach. Presence records for all species (including the species being modelled) were included as target group background points (Phillips et al., 2009). After filtering to one record per pixel we included 1101 background points in each model run. We evaluated the model's predictive performance using two threshold‐independent metrics using cross‐validation: the area under the Area Under the Curve (AUC; Jiménez‐Valverde, 2012) and the Boyce index (Hirzel et al., 2006). AUC values range from 0 to 1, where 1 indicates perfect ranking and 0.5 indicates a ranking by chance (Phillips & Dudík, 2008). The Boyce index ranges from −1 to 1, where values closer to 1 indicate that the predictions are consistent with the presence sites, and 0 values indicate predictions made by chance. In order to appropriately threshold the projections, fivefold cross‐validation was employed to determine the optimal threshold value that maximizes the sum of sensitivity and specificity (Liu et al., 2016). Predicted species’ distributions were then stacked by summing species’ thresholded projections in a given cell within their distribution types (cryophilic and cryotolerant) (Ferrier & Guisan, 2006); due to variable trend projections in cryophobic species, these models were presented separately (i.e., not stacked). Stacked projections were then converted to a binary measure indicating where at least one species of that type was predicted to occur.

Area calculations for all species (independently) and all projection scenarios (current and four future) were obtained for both probabilistic and thresholded projections. The area was calculated for both the full study area (northwards of 30°N) and northwards of every 10° increment between 40 and 80°N. The area occupied by a species was calculated as the sum of the predicted values in cells above a given latitude multiplied by the area of a single cell (43.15 km^2^ due to the North Pole Lambert Azimuthal Equal Area projection). Area calculations using thresholded projections are used for results in the main paper. Probabilistic model projections and area calculations for each species can be accessed via Figshare (https://doi.org/10.6084/m9.figshare.14751753.v6).

## 3 RESULTS

**FIGURE 2:**
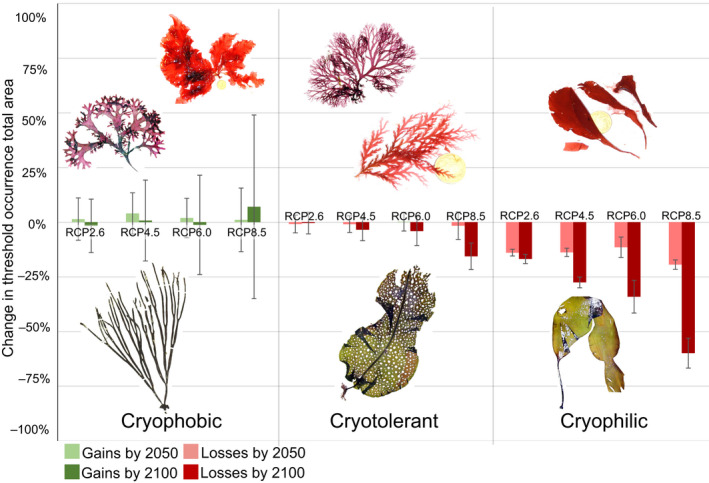
Total gain and/or loss in threshold occurrence area for the years 2050 and 2100 under Representative Concentration Pathway (RCP) scenarios (2.6, 4.5, 6.0, and 8.5), and according to three distribution types analysed for marine seaweeds. Cryophobic = Chondrus *crispus* (top left), *Delesseria sanguinea* (top right), and *Himanthalia elongata* (bottom); Cryotolerant = *Euthora cristata* (topmost), *Odonthalia dentata* (middle), *Agarum clathratum* (bottom); Cryophilic = Dilsea *socialis* (top), and *Laminaria solidungula* (bottom). Note, specimens are not to scale

**FIGURE 3:**
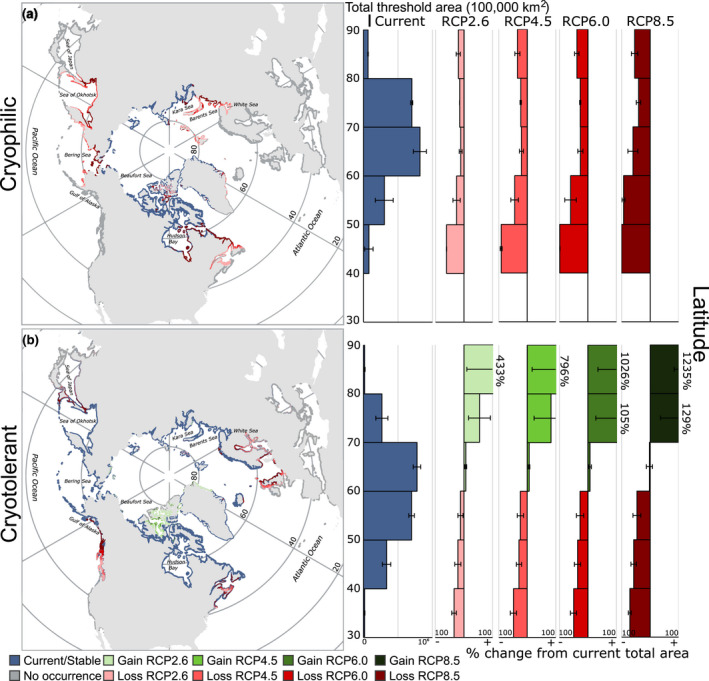
Stacked species projections under various Representative Concentration Pathway (RCP) scenarios by the end of the century (2100) in marine forest species (a) adapted to or (b) tolerant of the Arctic environment, including per cent gains and losses in total threshold area according to latitude. Present‐day threshold predicted occurrence is depicted in blue (stable) or red (future loss under climate change) and indicates the presence of at least one of the species modelled. All areas considered (coloured and dark grey) represent the environmental layers analysed, masked for depth (>100 m) or distance from shore (>10 km), and depositional substrate (sand/mud) as per Nyberg and Howell (2016). For individual species responses according to RCP scenarios, please refer to results provided on FigShare (https://doi.org/10.6084/m9.figshare.14751753.v6)

**FIGURE 4:**
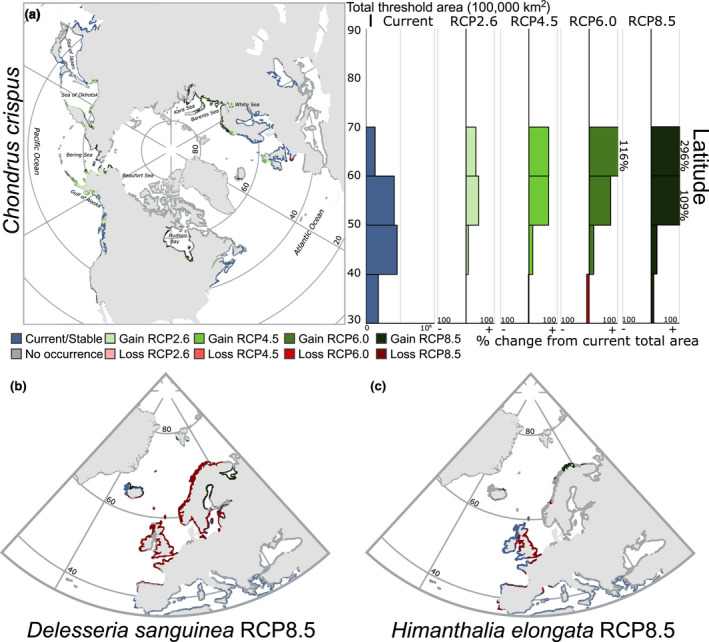
Species model projections under various Representative Concentration Pathway (RCP) scenarios by the end of the century (2100) in cryophobic marine forest species, including (a) *Chondrus crispus*, (b) *Delesseria sanguinea*, and (c) *Himanthalia elongata*. Per cent gains and losses in the total threshold area according to latitude are also presented for *Chondrus cripus*. Present‐day predicted threshold occurrence is depicted in blue (stable) or red (loss under climate change). All areas considered (coloured and dark grey) represent the environmental layers analysed, masked for depth (>100 m) or distance from shore (>10 km), and depositional substrate (sand/mud) as per Nyberg and Howell (2016). For individual species responses according to RCP scenarios, please refer to results provided on FigShare (https://doi.org/10.6084/m9.figshare.14751753.v6)

Stacked model projections according to the affinity for the Arctic environment showed contrasting responses to varying levels of future emissions (Figure 2). Contractions in the total amount of habitat were observed in cryophilic species, even by mid‐century under RCP 2.6. Losses in the total area occupied by cryophilic species were progressively accentuated with increasing emission scenarios, with a net loss of 67% of suitable habitat in *L*. *solidungula*, and a net loss of 53% of suitable habitat in *D*. *socialis*, both by 2100 under RCP 8.5 (Figures 2 and 3). Total suitable habitat was also lost under all RCP scenarios for cryotolerant species, although the per cent change in area was considerably less than the losses observed in cryophilic species (Figures 2 and 3). A net loss of 20% and 30% of total suitable habitat was observed in *A*. *clathratum* and *O*. *dentata* by 2100 under RCP 8.5, respectively, whilst a small net gain of 3% was observed in *E*. *cristata*. Cryophobic marine forest species featured highly varied projections. *Chondrus crispus* featured substantial increases in total area under all RCP scenarios, nearly doubling suitable habitat by 2100 under RCP 8.5 (90% increase; Figure 4a). Considerable losses were observed in *D*. *sanguinea* and *H*. *elongata*, with losses up to 24% and 45% by 2100 under RCP 8.5 (Figure 4b,c). Across all species, we achieved moderate to strong model performance, with AUC between 0.63 and 0.89 and Boyce index between 0.1 and 0.67 (Table S2).

MaxEnt models predicted the ubiquitous occurrence of cryophilic species throughout the Arctic environment, including predicted area >80°N under all RCP scenarios. At the southern range edge, cryophilic species showed severe contractions northwards (Figure 3a). Under RCP 2.6, losses were observed in Atlantic Canada, the Sea of Okhotsk and extending into the Bering Sea, on Svalbard, the White Sea, the Kara Sea and portions of the Canadian Archipelago (Figure 3a). Contractions continued northwards under progressively higher RCP scenarios, extending into Hudson Bay (Canada), the Barents Sea (Russia), the Sea of Okhotsk and the northernmost reaches of the Bering Sea by the end of the century under RCP 8.5. Other areas of suitable habitat for cryophilic species remained stable regardless of emission scenario, particularly along much of the Siberian coastline, the northern half of Greenland, and the majority of the Canadian Arctic Archipelago (Figure 3a).

Cryotolerant marine forest species showed modest expansions at northern latitudes, along with contractions at their southern range edge (Figure 3b). High‐latitude expansions were especially evident in the Arctic Archipelago and northern Greenland, corresponding to areas where suitable habitat was lost in cryophilic species, and occurring even under modest climate change (RCP 2.6; Figure 3b). Overall, habitat expansions in the north did not compensate for losses at the southern range edge of species (Figures 2 and 3b). Areas of predicted occurrence for cryotolerant species were generally stable across all RCP scenarios, with notable exceptions at the southern range edge. Cryotolerant species were predicted to contract northwards in waters surrounding Ireland and the UK, in much of Atlantic Canada, in the Gulf of Alaska and British Columbia (Canada) and in some areas of the Sea of Japan and the Sea of Okhotsk (Figure 3b).

Among the cryophobic marine forest species, the amphi‐Atlantic *C*. *crispus* showed substantial northern range edge expansions. Habitat expansions progressively increased with the RCP scenario under consideration, overlapping in some areas with the habitat lost in cryophilic and cryotolerant species (Figures 3 and 4). Expansions were prominent in the Gulf of Alaska, the Sea of Okhotsk, the Bering Sea, northern Norway and into the Barents, White and Kara Seas, and even in Hudson Bay under RCP 8.5. Cryophobic marine forest species did not generally encroach on areas predicted to be stable for cryophilic species under climate change. In contrast to *C*. *crispus*, substantial losses in predicted suitable habitat were observed in *D*. *sanguinea* and *H*. *elongata*, both restricted to European waters (Figure 4b,c), whilst gains in habitat under climate change were limited to some areas of Iceland, northern Norway, and the White and Baltic Seas.

## 4 DISCUSSION

Our objective was to estimate potential global gains and losses of northern hemisphere marine forests under climate change scenarios, particularly from the perspective of the Arctic environment. Our results highlight troubling declines forecasted for species endemic to the polar marine environment (Figure 2), but also suggest cryophilic marine forests may be currently more extensive in the Arctic than previously imagined (Figure 3a). Our results also indicate that succession from cryophilic to cryotolerant and cryophobic assemblages may occur within the Arctic, and that these ecosystem transitions may be unavoidable now for some areas (Figures 3 and 4).

### 4.1 The ubiquity of cryophilic marine forests in the Arctic

An important consideration for quantifying potential high latitude expansions under climate change is quantifying the hypothesized extent of contemporary marine forests adapted to the Arctic environment. Unfortunately, the Arctic has received far less attention compared to other areas of the globe when trying to arrive at range estimates (Melo‐Merino et al., 2020; Starko et al., 2021). For instance, Jayathilake and Costello (2021) recently boosted the estimated global extent of the kelp biome from 22% to 36% of the world's coastlines after adding Arctic records to their models, confirming an enormous amount of the kelp biome likely occurs at high latitudes. Müller et al. (2009) and Assis et al. (2018a) included the Arctic endemic kelp *L*. *solidungula* in distribution models, but their analyses were restricted to the North Atlantic and the adjacent Arctic regions, omitting circumpolar responses. Other seaweed taxa are also endemic to the Arctic, including phylogenetically distantly related red and green algae (Bringloe et al., 2020; Wilce, 2016; Wulff et al., 2011), yet these species remain largely overlooked in modelling efforts. Our results therefore present crucial context for evaluating the threat of climate change to the increasingly recognized endemic portion of the Arctic marine flora (Bringloe et al., 2020; Wilce, 2016).

Among the most surprising findings from our modelling was the predicted ubiquitous presence of cryophilic marine forest species at high Arctic latitudes in present‐day conditions. The northern ice‐pack has long been assumed to limit the northern range of marine forests given the need for an open water season for photosynthesis (Campana et al., 2009; Krause‐Jensen & Duarte, 2014; Müller et al., 2009). Persistent, multi‐year sea ice, however, is surprisingly limited along high latitude coastlines, straddling the western islands of the Canadian Archipelago and the northernmost coastlines of Greenland, often with scattered pockets of open water (polynas or leads; Cavalieri & Parkinson, 2012). Unfortunately, satellite observations beginning in 1979 do not predate 19th‐century warming, and the current declining trends in the extent and thickness of the northern ice pack (Cavalieri & Parkinson, 2012; Rigor & Wallace, 2004) are unlikely to reflect longer‐term perennial sea ice dynamics. It is, therefore, difficult to gauge whether marine forests in the Arctic were ubiquitous prior to, or as a result of, Anthropocene warming.

Marine forests were present at high Arctic latitudes prior to accelerated sea ice loss within the past two decades (Stroeve et al., 2012), as evidenced by collections from astonishing latitudes during the 20th century. For instance, Lee (1973) describes dredging seaweeds from a polynya at nearly 78^o^N between Brock Island and McKenzie King Island, pulling up Arctic endemic species such as *L*. *solidungula* (modelled here). Lee also collected *L*. *solidungula* from a small ice‐free area in the Robeson Channel at 82.4^o^N, whilst Lund (1951) reported on marine flora sampled from Jörgen Brönlunds Fjord in northeastern Greenland from 82^o^N. Assemblages at these latitudes were not exclusively cryophilic species, but also included some cryotolerant species not modelled here, for instance, the red alga *Coccotylus truncatus* (Pallas) M.J. Wynne & J.N. Heine and kelp such as *Saccharina latissima* (Linnaeus) C.E. Lane, C. Mayes, Druehl, & G.W. Saunders. These reports from areas typically regarded as inhospitable due to ice‐pack lend confidence to our range projections (Figure 3a). Indeed, of the environmental variables used to train our models, the presence of sea‐ice best predicted the presence of cryophilic species *D*. *socialis* and *L*. *solidungula* (Table 1), the latter of which is known to complete much of its annual growth under sea‐ice (Chapman & Lindley, 1981; Dunton & Schell, 1986). The importance of sea‐ice in modelling Arctic kelp distributions is also reported by Goldsmit et al. (2021). It can be expected, however, that contemporary marine forests at the highest latitudes are extremely patchy, persisting only where the ebb and flow of sea ice dynamics facilitate a brief growing season, and frequently occurring at low per area biomasses, depth ranges, and cover compared to marine forests at lower latitudes (Filbee‐Dexter et al., In press; Krause‐Jensen et al., 2012). Diminutive, poorly studied, and potentially more resilient alternate life‐history stages (e.g. kelp gametophytes) also occupy the Arctic benthos (Küpper et al., 2016), meaning ascertainment bias plays a large role in how we currently interpret patch dynamics (both in the Arctic and otherwise). As far as we know, there have been no efforts to extensively sample seaweeds at the highest latitudes reported here (80‐82^o^N), meaning the true extent of marine forests bordering the multi‐year ice pack remains unknown. Evidently, much of our ignorance on the northern range edge of marine forests in the Arctic stems from the inherent difficulties of conducting surveys at high latitudes.

**TABLE 1:** Per cent variable contributions towards present‐day MaxEnt models in species of marine macroalgae

Species	Mean salinity	Lt. min ice thickness	Lt. max ice thickness	Lt. min temp.	Lt. max temp.
Cryophobic					
*Chondrus crispus*	9.9	0	**14.1**	2.4	**73.5**
*Delesseria sanguinea*	**46.8**	0	2.9	**46.3**	4
*Himanthalia elongata*	**33.3**	0	30.5	**34.5**	1.6
Cryotolerant					
*Agarum clathratum*	**66.9**	0	5.3	**17.7**	10.1
*Euthora cristata*	**56.2**	5.5	**20.3**	7.5	10.5
*Odonthalia dentata*	**45.9**	0.2	**18.5**	23.7	11.6
Cryophilic					
*Dilsea socialis*	0.1	0.1	**91.8**	**5**	3
*Laminaria solidungula*	0	0	**88**	0.1	**11.9**

Note

Lt, Long term (average of yearly values, 2000–2014). The top two variables in terms of per cent contributions to the MaxEnt models are bolded for each species.

### 4.2 Expansions of marine forests in the Arctic under climate change

Gains and/or succession in Arctic marine forests under climate change have the potential to be substantial. For instance, Lantuit et al. (2012) indicate that 34% of the world's coastline is affected by Arctic permafrost, a reflection of the vast amount of high latitude coastline that will potentially become more productive under climate change. As much as 340,658 km^2^ of potential habitat for macrophytes currently exists in the Arctic (Krause‐Jensen et al., 2020). Time‐series data for the past 60–70 years suggest that suitable habitat in the Arctic has increased by 8.1% for intertidal algae and by 44.6% for subtidal algae, with the largest increases observed in Svalbard, though these per cent increase estimates did not account for substrate requirements (Krause‐Jensen et al., 2020). Medium confidence (IPCC+confidence scale of 5–8 in 10 chance of being correct) was placed on the projection that macrophytes are expanding in the Arctic, but this included within range increases in abundance as well as total gains in habitat area. Ecological niche modelling also supports this trend of expansion continuing into the future depending on the severity of climate change, with substantial gains in the Arctic projected for many canopy‐forming brown seaweeds (Assis et al., 2018; Goldsmit et al., 2021; Jueterbock et al., 2013, 2016), and similar trends of northward shifts reported in temperate North Atlantic taxa (Westmeijer et al., 2019). Modelling work, however, has focused largely on cold‐temperate taxa that extend into the Arctic basin (e.g. Assis et al., 2018a; Krause‐Jensen et al., 2020; Wilson et al., 2019), without considering distinct global responses of cryophilic (i.e., endemic) or cryotolerant marine forest species.

Our analysis indicates that the potential for high latitude expansions of marine forests depends on the relative affinity of species for the contemporary Arctic environment. As discussed above, cryophilic species likely do not have a present‐day northern range limit, meaning there is likely limited uncolonized habitat in the Arctic to expand into as climate change progresses (Figure 3a). As the only possibility within our models was to contract northwards, habitat losses under climate change were severe in these species, even under modest climate change (Figures 2 and 3). Exceptions to this trend may be small, unstudied areas of Arctic coastal zones where persistent multi‐year sea ice exists, but is rapidly disappearing (e.g. Pope et al., 2012). As well, species may respond to warmer waters by shifting with deepening isotherms (Chaikin et al., 2022; Davis et al., 2021), an aspect not captured in our models, though the depth of the photic layer presents a major constraint on this possibility for photosynthetic organisms (Jorda et al., 2020). The local extirpations projected here may therefore represent significant rearrangements of the benthos rather than true losses. Meanwhile, our results support the hypothesis of future high Arctic expansions for cryotolerant species and limited lower‐latitude expansions of cryophobic species, mainly in areas where cryophilic species are predicted to become extirpated in the future (Figures 3 and 4). High‐latitude expansions were inconsistent, however, in the cryophobic species modelled here, with substantial gains observed in *C*. *crispus*, and erratic and substantial losses in *H*. *elongata* and *D*. *sanguinea*. These results speak to potentially variable responses in species tailored to cold‐temperate waters. A possible explanation for this is responses in more widely distributed species (proxied here in *C*. *crispus*) are driven by less dynamic environmental preferences (e.g. temperature alone; Table 1), whereas species with limited distributions are tailored to localized conditions (i.e., the intersection of several factors such as temperature and salinity; Table 1), thus inhibiting their potential to expand into northern environments as climate change progresses. Thus, we cannot expect ubiquitous future responses in the gains and losses of marine forest species. Nonetheless, our results provide context for understanding the potential of future marine forest expansions in the Arctic; at the community level, the high Arctic may witness a shift away from present‐day dominance by Arctic endemic species towards greater inclusivity of cryotolerant species under climate change, with a potential succession of cryophobic species at lower Arctic latitudes. Such changes in community composition are potentially reflected in Arctic areas under the influence of warm Atlantic water, such as Svalbard (Fredriksen et al., 2014; Hop et al., 2012). Discussions surrounding novel gains in marine forest habitat in the Arctic, however, are incompatible with the already ubiquitous presence of cryophilic species in the north.

Several factors may limit northward expansions predicted here, particularly in cryophobic species tailored to temperate conditions, or potential gains in overall productivity for those species already ubiquitous in the Arctic basin. For instance, a longer ice‐free season has not led to increased production in the kelp *L*. *solidungula* along Alaska's northern coastline, as increased access to light was nullified by wind‐driven resuspension of sediment (Bonsell & Dunton, 2018). These results suggest annual growth of marine forests in the Arctic may depend on the brief (1–3 weeks) period surrounding ice break‐up when sea‐ice can reduce wind and subsequent water column turbidity. The role of turbidity in Arctic marine forest productivity could become increasingly important in the coming decades as permafrost melt and coastal erosion release more particulate organic material into the coastal zone (Fritz et al., 2017; Gagarev et al., 2019; Paar et al., 2016). Biological factors such as grazing pressures by fauna moving north with declining ice cover could also play an important role in determining marine forest productivity and biomass in the Arctic (Blicher et al., 2007; Krause‐Jensen et al., 2020). Propagule dispersal may also limit the movement of species further into the Arctic basin, particularly where cold water barriers impede population movement/establishment. For instance, habitat gains by cryophobic species in the Arctic basin were discontinuous, specifically in Hudson Bay, an area where propagules would have to pass through unsuitable habitats in northern parts of Labrador and Quebec (Canada) to reach habitable waters under climate change (Figure 4a). The light regime is also an important consideration for expansions into the Arctic basin, as the polar day and night exceed 24 h at latitudes above the Arctic circle (66.5°N) surrounding the yearly solstices. Day length is an important trigger for physiological responses such as reproduction in seaweeds, and some species anticipate extremes in year‐round conditions in the Arctic (Wienke and Amsler, 2012). Species expanding further north in the Arctic basin are therefore expected to be opportunistic, tolerating wide ranges in light regime, whilst others may have their expansions north constrained due to reduced reproductive success under continuous light (e.g. *Alaria esculenta*; Martins et al., 2022). Moreover, the integration of phenological information is known to adjust projections of future ranges of seaweeds (Chefaoui et al., 2019). We therefore caution that gains in suitable habitat predicted here will not necessarily equate to realized range expansions. As with most things related to climate change, predicting the responses of biological communities is complex, and strategic future monitoring will be crucial to validate the projections outlined here.

### 4.3 Loss and succession in Arctic marine forests

Overall, losses in total suitable habitat were predicted to occur for taxa adapted to, or tolerant of, the contemporary Arctic environment (i.e., cryophilic and cryotolerant species; Figure 2). As described above, for species that are already distributed throughout the Arctic basin, there will be little to no gain in suitable habitats at high latitudes, meaning there is no way to compensate for potential losses at their southern range edge. Similar losses under climate change at the warm range edge of seaweeds have been predicted for numerous species (Assis et al., 2018; Goldsmit et al., 2021; Jueterbock et al., 2013, 2016; Martínez et al., 2018; Westmeijer et al., 2019) and has already been documented in some areas of the globe (Vergés et al., 2016). Losses in cryophilic and cryotolerant marine species were not restricted to particular latitudes (Figure 3a,b), but rather corresponded to seawater isotherms. Arctic waters (i.e., those with a minimum bottom temperature of <0°C) reach as far south as 50^°^N in Canada (Gulf of St. Lawrence and Newfoundland), whilst relatively warm Atlantic waters reach 70^°^N in Europe, creeping over the northern coast of Norway (Figure 1a; Assis et al., 2018b). Species distributions of seaweeds have long been understood within the context of marine surface isotherms (Lüning, 1990; Müller et al., 2009), and recent modelling confirms the importance of temperature regimes as an explanatory variable (e.g., Martínez et al., 2018). Müller et al., (2009) predicted similar losses in Arctic marine forests solely on the basis of changes to sea surface temperature, including the loss of *L*. *solidungula* from Hudson Bay, southern Baffin Island, Newfoundland and Labrador, and coastlines of the Kara and Barents Seas (Russian Island Novaya Zemlya). Despite severe overall losses, there appears to be little risk of contemporary Arctic marine forests becoming extirpated, even under the most extreme climate projections at the time scales considered here (Figure 2), though longer time scales remain unassessed (Lyon et al., 2022). Ecological investigations of intertidal communities on the West coast of Greenland also support the idea that Arctic marine biota will remain resilient to future climate change (Thyrring et al., 2021).

An important consideration for the loss of marine forest populations is their genetic diversity, which may act as a proxy for their resilience to climate change and acute events such as marine heatwaves. For instance, Wernberg et al. (2018) showed that genetic diversity consistently explained, to a large extent, the responses of Australian kelp forests to marine heatwaves. Low‐diversity populations at the warm range edge were eliminated, whilst mid and high diversity populations remained partially or fully intact, respectively. Unfortunately, our understanding of genetic diversity in northern hemisphere marine forests is largely limited to the analysis of organellar sequences, and non‐existent at the genomic level. Genetic diversity is expected to be high at lower latitudes where refugia are hypothesized to have occurred during the last glaciation (~21 ka). However, recent work in the North Atlantic (Bringloe et al., 2020; Guzinski et al., 2020) and North Pacific (Grant & Bringloe, 2020; Grant et al., 2020) have challenged this paradigm by suggesting genetic diversity is also high in some northern populations. Conducting genomic surveys of marine forest populations would provide definitive context for anticipating the resilience of populations in threatened areas identified here (Figure 2). In particular, genomic surveys are needed for cryophilic and cryotolerant marine forest species at their southern range edge, specifically in Atlantic Canada, the UK, Norway and into the Barents Sea, Pacific Canada into the Gulf of Alaska, and the Bering Sea and the Sea of Okhotsk.

The cryophobic species *C*. *crispus* was predicted to respond favourably to climate change by greatly expanding its overall distribution (Figure 4a). Unlike cryophilic and cryotolerant flora, predicted losses at the southern range edge under climate change either did not occur or were outpaced by gains at northern range edges (Figure 4a), leading to profound net gains in habitat. These gains reached quite deep into the Arctic basin as defined by AMAP (1998), particularly in the Barents Sea, the northern Bering Sea, and parts of Hudson Bay (Figure 4a). These gains in suitable habitat also generally corresponded to areas of the globe where cryophilic or cryotolerant species become extirpated at their southern range edge under climate change (Figure 3a,b). Thus, certain areas of the globe may experience some level of a succession of Arctic associated taxa with temperate species depending on the emissions scenario, a borealization of the Arctic already reported through Atlantic (Asbjørnsen et al., 2020; Csapó et al., 2021) and Pacific (Polyakov et al., 2020) pathways. Unfortunately, some of the areas with predicted succession correspond to locations of subsistence for human societies (Figures 3 and 4). For instance, aquaculture developments in the Gulf of Alaska (Stekoll, 2019) ought to consider the stability of cold‐adapted species such as kelp to climate change effects. Stakeholders will have to adapt or move with the biota. Inuit in the Qikiqtaaluk region (Baffin Island) use *L*. *solidungula* as traditional medicine (Black et al., 2008), suggesting its presence may be important for supporting identities and cultural practices. On the contrary, cryotolerant and cryophobic species such as *S*. *latissima* and *L*. *hyperborea*, respectively, are likely to experience habitat gains in some Arctic locations and are among the most commercially exploited or farmed kelps in the northern hemisphere, potentially providing new opportunities for northern communities (Buschmann et al., 2017).

### 4.4 Limitations of the models

Our projections are not without important caveats and avenues for improvements moving forward. To start, model performance was subpar in the cryotolerant species (AUC values of 0.63–0.72) and did not improve when adding other environmental variables such as nutrient concentrations. This appeared to be partly driven by broad geographic distributions capturing a wide range of environmental conditions (Figure 1c). Thus, whilst model performance might improve with the right set of environmental layers, it also seems likely these species are inherently difficult to model due to their wide biological preferences. We also note that the Boyce Index is the preferred metric for presence‐only datasets and we achieve scores of ~0.4 or higher for all but one species. The low Boyce Index score for *D*. *sanguinea* (0.1) was not reflected in the AUC score (0.81), and we note that Boyce Index is susceptible to poor performance on species with low prevalence/small sample size (Hirzel et al., 2006), as was the case in *D*. *sanguinea*. Taxonomic misidentifications may have further impacted model performance. Whilst species were carefully selected on the basis of confident taxonomic IDs, it is possible Northwest Pacific records for *E*. *cristata* and *A*. *clathratum* were conflated with congeners from this diverse region with limited survey information. The reliability of the occurrence records as a single, cohesive species with consistent niche preferences should always be scrutinized if considering the individual species models, and updated in light of genomic level information (e.g., Bringloe et al., 2021). Besides confirming the true extent of species, such data would add further insight on the adaptive capacity of species under climate change. As reported by Hu et al. (2021), intraspecific genetic variation provides an important context for predicting the distribution of marine species.

Projections also did not necessarily reflect individual species distributions (e.g., Atlantic *C*. *crispus* in the Pacific) or accurately reflect distributions from hypo‐ and hyper‐saline environments (e.g., the Baltic and Mediterranean Seas). Other environmental variables with future projections (e.g., nutrient concentrations) and/or limiting the geographic scope of analysis are needed to refine species projections in these instances. Note, we were interested in the species modelled insofar as they proxied different affinities to the Arctic environment, not the individual species themselves, thus we were willing to accept some level of inaccuracies in the species models. Furthermore, the time frame for the Bio‐Oracle layers (2000–2014) is misaligned with the majority of the records used to train our models (~1900 onwards, with 94% of records postdating 1950 but only 15% postdating 2000). Assuming climate change has appreciably shifted marine climatic regimes since 1900, upper‐temperature limits are potentially biased in the model training, thus leading to a slight shift in the projected latitudinal ranges and conservatives estimates of habitat loss at the southern range edge of cryophilic and cryotolerant species. Moreover, by stacking our models in cryophilic and cryotolerant species, our interpretations are necessarily sensitive to the presence or absence of a single species. Readers can cross‐check the individual species projections, including older models with alternative species, to assess the consistency of trends (https://doi.org/10.6084/m9.figshare.14751753.v6). We also emphasize that area estimates of suitable habitat must be carefully interpreted. Though internally consistent, these estimates are not directly comparable to other values published in the literature, nor should they be employed in further calculations without carefully considering the analytical choices made here. This is because any occurrence within the environmental grids was counted as the full reprojected area size of 43.15 km^2^, thus likely inflating our estimates given smaller scale heterogeneity could not be captured. Similarly, this may have led to overestimates of suitable Arctic habitat at the northern range edge, especially if marine forests are patchier further north, as we hypothesized here. Setting the max depth of our environmental grids to 100 m also likely overestimated habitat amounts. As stated previously, incorporating higher‐resolution spatial data linked to depth information would enhance the accuracy of predicting climate change responses (e.g. Davis et al., 2021). On the contrary, our decision to mask the environmental layers for depositional versus erosional shorelines may have underestimated the amount of Arctic habitat available to marine forests. The northern shorelines of Alaska, which were mostly excluded from our models, are known to host patches of marine forests interspersed among sediment‐laden bays (Wilce & Dunton, 2014), and substrate requirements may be less strict in some species (i.e., Lee (1973) reports on seemingly healthy unattached communities in calm bays, whilst Filbee‐Dexter et al. (In press) show that kelp forests in the East Canadian Arctic are also present in sedimentary habitats).

We recognize that throughout our manuscript, we have simplified arguments and interpretations in an attempt to synthesize broad‐scale understanding. For instance, stacking models was done to facilitate interpretations at a broader taxonomic/ecological niche scale, although this came at the expense of considering heterogeneity in species responses within the distribution types investigated. Similarly, emission scenarios were used as a proxy for climate change severity, at the expense of recognizing localized shifts in climate (both temporally and spatially). Our interpretations, therefore, do not preclude the possibility of localized reversals in climate trends, ecosystem responses, and refugia for cold‐adapted species (particularly if taxa shift to deeper waters). The reader should also consider the errors inherent in producing models from higher latitudes where environmental parameters are often interpolated, and in projections under future scenarios where novel environmental circumstances may not be reflected in models trained on contemporary conditions. We therefore emphasize that the predictions presented here are hypotheses (as is the case with any future projections) and that the skill of the underlying models remains to be assessed (Stow et al., 2009) and is expected to be variable for the Arctic environment (Séférian et al., 2020).

### 4.5 Conclusions

The potential for northward range expansions of marine forests under climate change has long been hypothesized, however, the focus has remained on temperate species and locations. Here, we have provided a global synthesis of predicted changes to Arctic coastal marine forests under climate change, and highlight overlooked aspects regarding Arctic adapted species, in particular the ubiquity of endemic species at high latitudes. Our work has important implications for how Arctic marine coastal ecosystems are perceived, both in terms of their unique composition and stability under future climates. Climate change will continue to threaten Arctic endemic taxa at their southern range edge, where the balance of marine coastal communities may tip towards temperate marine forests, regardless of the severity of warming. Among the key considerations moving forward is whether the replacement of cryophilic or cryotolerant species by cryophobic taxa will result in changes to ecosystem services and whether these changes are potentially buffered by more resilient native species. Direct comparison of the services provided by canopy forming kelp (e.g. *L*. *solidungula* versus *A*. *clathratum*) and understory red algae (e.g. *D*. *socialis* versus *E*. *cristata*, and *O*. *dentata*) would provide a crucial context in this regard. Meanwhile, routine survey sites (e.g. Bartsch et al., 2016) will continue to provide real‐time insights on regime changes and their ecological consequences. Despite overall losses in habitat extent, Arctic marine forests will persist throughout their northern range. Undoubtedly, novel discoveries about the Arctic marine biota remain to be unearthed, discoveries that can hopefully continue under the Anthropocene climate.

## CONFLICT OF INTEREST

No potential conflict of interest was reported by the authors.

## AUTHOR CONTRIBUTIONS

TTB, DPW, and HV conceived the study. TTB, JG, AMS, KFD, KAM, CWM, and KLH generated data. DPW conducted the analyses. TTB, DPW, and JG wrote the manuscript. DPW, HV, JG, AMS, KFD, KAM, KLH, and CWM edited and revised the manuscript.

## Supporting information

Table S1

Table S2

## Data Availability

All occurrence records for model training, species‐specific projections for the current day and RCP scenarios, and area calculations (present and future) are available on Figshare: https://doi.org/10.6084/m9.figshare.14751753.v6. The modelling workflow can be accessed via GitHub (https://github.com/Doi90/arctic_marine_forest), and additional details regarding methodology standardized according to the ODMAP protocol (Zurell et al., 2020) are provided in the supplementary material.
